# Supplementary material for: Population Pharmacokinetics of Colistin Methanesulfonate Sodium and Colistin in Critically Ill Patients: A Systematic Review
Source: Pharmaceuticals (Basel). 2021 Sep 6;14(9):903. doi: 10.3390/ph14090903 (PMC8472798; doi:10.3390/ph14090903)
Supplement: Supplementary file 1 [file pharmaceuticals-14-00903-s001.zip › pharmaceuticals-1333649-supplementary.pdf]

pharmaceuticals

MDPI

Supplementary Materials

Table S1. Characteristics of included studies.

Author, year	Study size	Population	Age (year)	Body weight (kg)	CrCl (ml/min) (renal impaired patients)[a]	Brand	Preparation[b]	Loading dose	Sample Collection		Bioanalytical analysis
									First dose	Repeated dose	
Markou et al., 2008 [20]	14	ICU Patients	62±19.2	72.5±8.5	109.6±38.1 (1 patient)	Colistin	30,000IU/ml (100ml)	No	No	Yes	LC-Fluorimetry
Plachouras et al., 2009 [9]	18	ICU Patients	63.6±11.03	82.2±10.8	82.3±24.4 (2 patients)	Colistin	30,000IU/ml (100ml)	No	Yes	Yes	LC-MS/MS
Imberti et al., 2010 [23]	13	ICU Patients	45.3±15.4	81.4±10	125.4±19.8	Colimicina	40,000IU/ml (50ml)	No	No	Yes	LC-Fluorimetry
Garonzik et al., 2011 [6]	105	ICU Patients	63.25±14.5	63.65±15.1	56.6±33.5 (98 patients)	Colistate	Not reported	No	No	Yes	LC-Fluorimetry
Karvanen et al., 2012 [21]	5	ICU patient (CVVHDF)	66±6	78±8	79±26.5 (5 patients)	Colistin	20,000IU/ml (100ml)	No	Yes	Yes	LC-MS/MS
Mohamed et al., 2012 [10]	10	ICU Patients	55.4±10.28	81±12.6	113±36.8 (1 patients)	Colistin	10,000-60,000IU/ml (100ml)	Yes	Yes	Yes	LC-MS/MS

CrCl: Creatinine clearance; GSK: GlaxoSmithKline; ICU: Intensive care unit; IU: International unit; IU/ml: International unit/milliliter; kg: Kilogram; LC: Liquid chromatography; MS/MS: Tandem mass spectrometry mg: Milligram; MIU: Million International unit; ml/min: Milliliter/minute; min: Minute. [a]Renal impaired patients, CrCl < 50ml/min. [b]The solution is considered stable when the concentration is >80, 000IU/ml.

Table S1. Characteristics of included studies (continue).

Author, year	Study size	Population	Age (year)	Body weight (kg)	CrCl (ml/min) (renal impaired patients)[a]	Brand	Preparation	Loading dose	Sample Collection		Bioanalytical analysis
									First dose	Repeated dose	
Karnik et al., 2013 [12]	15	ICU Patients	27.3±6.72	61.4±5.5	123.5±38 (1 patient)	Xylistin™	40,000IU/ml (50ml)	No	Yes	Yes	LC-MS/MS
Grégoire et al., 2014 [11]	73	ICU Patients	58.25±15.3	91.7±28.3	138.5±74.2	Colimycine	40,000IU/ml (50ml)	No	Yes	Yes	LC-MS/MS
Matthieu et al., 2014 [15]	12	ICU Patients	54±19	79±17	134±53	Colimycine	40,000IU/ml (50ml)	No	No	Yes	LC-MS/MS
Karaiskos et al., 2015 [3]	19	ICU Patients	56.2±17.7	77.6±16.4	92.1± 54 (6 patients)	Colistin	45,000IU-[b]90,000IU/ ml (100ml)	Yes	Yes	Yes	LC-MS/MS
Jacob et al., 2015 [22]	8	ICU patient (HD)	64.8±8.3	77±11.5	24.4±6.8 (8 patients)	Colimycine	30,000IU/ml (50ml)	Yes	Yes	Yes	LC-MS/MS
Leuppi-Taegtmeyer et al., 2019 [14]	10	ICU patient (CRRT)	50.4±16	65.7±11.6	Not reported (10 patients)	Not reported	Not reported	Yes	No	Yes	LC-MS/MS

CrCl: Creatinine clearance; GSK: GlaxoSmithKline; ICU: Intensive care unit; IU: International unit; IU/ml: International unit/mililiter; kg: Kilogram; LC: Liquid chromatography; MS/MS: Tandem mass spectrometry mg: Milligram; MIU: Million International unit; ml/min: Milliliter/minute; min: Minute. [a]Renal impaired patients, CrCl < 50ml/min. [b]The solution is considered stable when the concentration is >80, 000IU/ml.

Table S1. Characteristics of included studies (continue).

Author, year	Study size	Population	Age (year)	Body weight (kg)	CrCl (ml/min) (renal impaired patients)[a]	Brand	Preparation	Loading dose	Sample Collection		Bioanalytical analysis
									First dose	Repeated dose	
Moni M, et al., 2020 [5]	20	ICU Patients	55±13.5	65.1±6.7	115±23.9	Co-ly-monas ®	30,000IU/ml (100ml)	Yes	Yes	Yes	LC-MS/MS
Kristof-fersson et al., 2020 [2]	349	ICU Patients	66 ±16	Not reported	201.75±111.4 (54 patients)	Colimy-cine	Not reported	Yes	Yes	Yes	LC-MS/MS
Ram, et al., 2020 [4]	30	ICU Patients	39.3±14.2	61.7±11	95.9±12.6	Xylistin™	20,000IU/ml (100ml)	No	No	Yes	LC-Fluorimetry

CrCl: Creatinine clearance; GSK: GlaxoSmithKline; ICU: Intensive care unit; IU: International unit; IU/ml: International unit/milliliter; kg: Kilogram; LC: Liquid chromatography; MS/MS: Tandem mass spectrometry mg: Milligram; MIU: Million International unit; ml/min: Milliliter/minute; min: Minute. [a]Renal impaired patients, CrCl < 50ml/min. [b]The solution is considered stable when the concentration is >80, 000IU/ml.
